# Memory like NK cells display stem cell like properties after Zika virus infection

**DOI:** 10.1371/journal.ppat.1009132

**Published:** 2020-12-28

**Authors:** Weshely Kujur, Oscar Murillo, Raju S. R. Adduri, Ramakrishna Vankayalapati, Nagarjun V. Konduru, Sachin Mulik

**Affiliations:** 1 Department of Pulmonary Immunology, Center for Biomedical Research, The University of Texas Health Science Center at Tyler, Tyler, TX, United States of America; 2 Department of Cellular and Molecular Biology, Center for Biomedical Research, The University of Texas Health Science Center at Tyler, Tyler, TX, United States of America; INRS - Institut Armand Frappier, CANADA

## Abstract

NK cells have been shown to display adaptive traits such as memory formation akin to T and B lymphocytes. Here we show that Zika virus infection induces memory like NK cells that express CD27. Strikingly, these cells exhibit stem-like features that include expansion capacity, self-renewal pathway, differentiation into effector cells, longer telomeres and gene signature associated with hematopoietic stem cell (HSC) progenitors. This subset shared transcriptional and epigenetic changes with memory CD8 T cells, stem cells and stem like T cells. These NK cells with memory and stem cell features, which we term “NK memory stem cells”, demonstrated greater antiviral potential than CD27^-^ or naïve CD27^+^ NK when adoptively transferred to Zika infected mice. Our results also suggest a role for the transcription factor TCF-1 in memory and stemness features of this NK subset. This study defines a unique TCF1^hi^ CD27^+^ NK subset with memory capacity and stem cell features that play a role in antiviral immunity.

## Author summary

The CD8 T cells exhibiting stem cell like properties occur in chronic viral infection and tumors and those stem like T cells respond to PD-1 blockade undergoing proliferative burst resulting in viral clearance and tumor immunity. It is unclear whether innate immune cells such as NK cells contain a subset with stem cell features and memory capacity. In the present study, using a mouse model of Zika virus infection, we identify memory like NK responses to Zika virus. The detailed transcriptomics, chromatin accessibility analysis and functional studies showed that these memory like NK cells also presented stem cell-like properties and shared signatures with memory CD8 T cells, stem cells and stem like T cells from chronic infection and tumors. These cells also demonstrated greater antiviral potential than naïve CD27^+^ NK cells when adoptively transferred to Zika infected mice. Our work highlights stem cell memory features in NK cells that are beneficial in fighting viral infection.

## Introduction

Natural killer (NK) cells play roles in clearance of virus infected cells and tumor surveillance and in the production of cytokines that orchestrate adaptive immune responses. Humans lacking NK cells suffer from life-threatening herpes virus infections [1,2] and are more susceptible to some cancers [3] thus highlighting the importance of NK cells. Traditionally NK cells are considered part of the innate defense mechanism but several recent reports indicate that NK cells can also express immune memory responding more effectively to secondary stimulation just like cells of the adaptive immune system [4–7]. The immunological memory phenotype of NK cells involves robust secondary expansion to haptens, cytokines or microbes. Murine NK cells possessing an activating receptor, Ly49H, undergo robust expansion, contraction, and memory formation after mouse cytomegalovirus (MCMV) infection [5]. Ly49H+ NK cells recognize infected cells expressing the MCMV-encoded protein, m157. Following infection with MCMV lacking m157, memory NK cell responses were absent. Memory NK cells provided more effective protection against MCMV or responded better to haptens than did naïve NK cells [5,7].

The CD8 T cell compartment harbors a subset that exhibits memory and stem cell properties [8–10]. These stemness properties include long term quiescence, self-renewal and multipotent ability to generate memory as well as terminal effector cells. Such subsets also occur in chronic viral infections [11–13] as well as in tumors [14–17] and were found to undergo a proliferative burst after PD-1 blockade therapy. Whether innate immune cells such as NK cells include a similar stem cell memory subset and contribute to anti-microbial defense is not known.

The subset of NK cell that exerts memory may express some properties shared with memory CD8 T cells [18] and as we show in this report several properties which are also characteristic of self-renewing hemopoietic stem cells (HSC). Indeed, memory like NK cells share some properties with progenitor exhausted memory CD8 cells (aka stem like CD8 T cells) found in chronic viral infections [11–13] and some cancers [14–17]. In this report we fully characterize what we refer to as “NK memory stem cells” (NK_SCM_) and show that such cells can act effectively to control Zika virus (ZIKV) infection in a mouse model system. Moreover, unlike terminally exhausted CD8 T cells, transcriptomics and epigenetic landscape of NK_SCM_ cells indicate that these cells appear resistant to developing functional exhaustion.

## Results

### Memory like NK response to ZIKV infection

**Fig 1:**
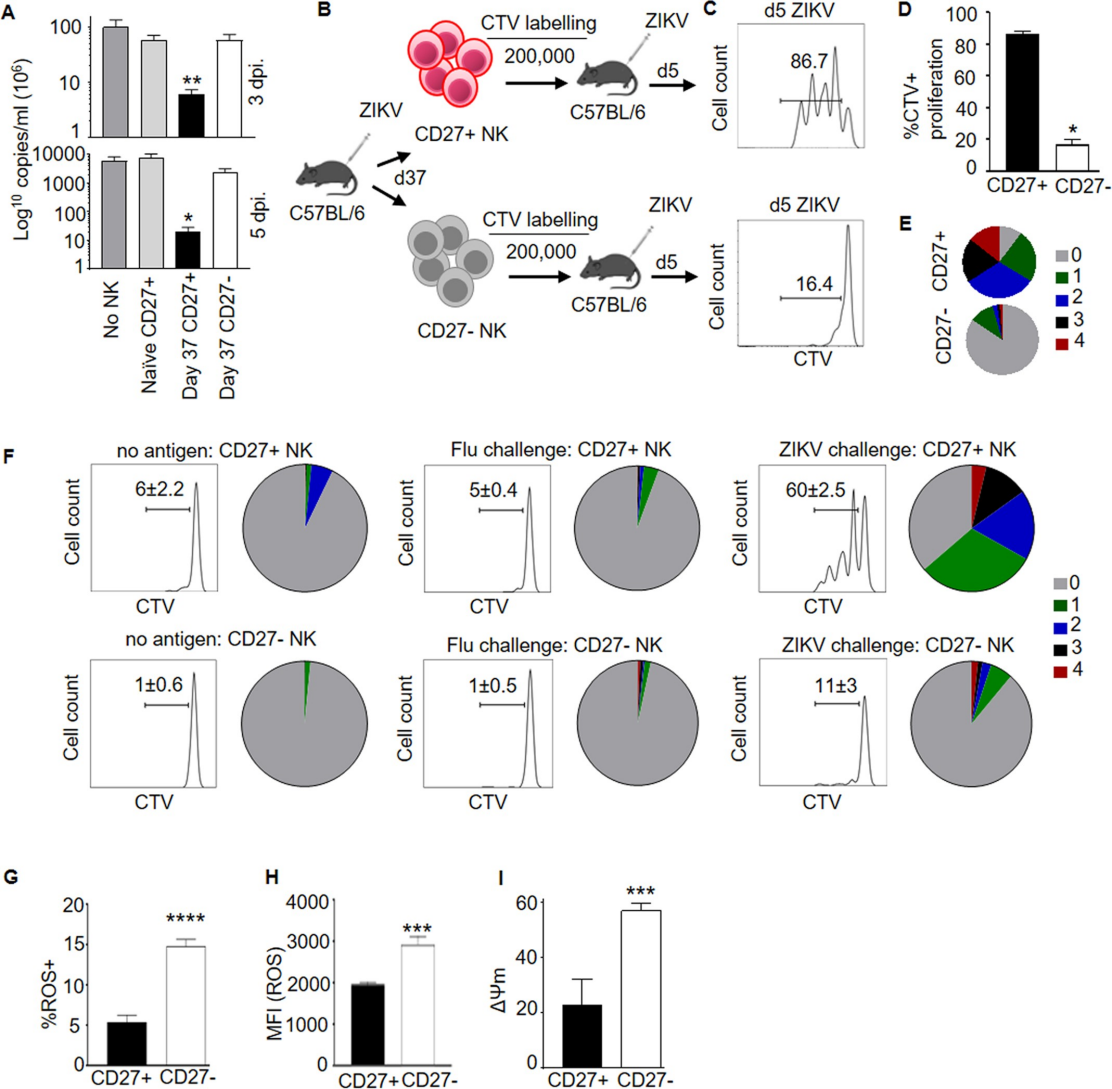
Memory like NK responses after Zika virus infection. (**A**) Viremia levels after transfer of naïve CD27^+^ NK, memory phase CD27^+^ or CD27^-^ NK in *Ifnar1*^-/-^ mice which were infected with ZIKV. Data are representative of 3 experiments (n = 6–8 per experiment). (**B**) Experimental setup depicting transfer of CTV labelled CD27^+^ or CD27^-^ NK cells into mice which were challenged with ZIKV and cells were analyzed 5 days later in spleen. (**C-E**) Cell division of respective NK cell populations in spleen of ZIKV infected mice. Data are representative of 3 experiments (n = 3–4 per experiment). (**F**) Cell division of memory phase CD27^+^ NK transferred into mice which were challenged either with ZIKV, *Flu* virus or no antigen (PBS). Data are representative of 2 experiments (n = 3 per experiment). (**G, H**) Analysis of ROS production in memory phase CD27^+^ or CD27^-^ NK. Data are representative of 3 experiments (n = 3 per experiment). (**I**) Loss of mitochondrial membrane potential in memory phase CD27^+^ or CD27^-^ NK. Data are representative of 2 experiments (n = 3 per experiment). Mean ± s.d. two-sided Student’s t-test, ANOVA. two-sided Student’s t-test, ANOVA. *P ≤ 0.05, **P ≤ 0.01, ****P ≤ 0.0001.

NK cell response to virus infection is compromised in the absence of type I interferons (IFNs) [19–21]. Thus, we searched for the presence of NK_SCM_ cells to ZIKV in interferon sufficient C57BL/6 mice, which initially show low viremia and cleared ZIKV without showing apparent clinical disease. To this end, C57BL/6 mice were infected with ZIKV (i/p, 10^7^ PFU) and changes in NK subtypes were recorded in spleen. While several NK receptors remain unchanged at day 8 post infection (p.i.), NK cells upregulated CD44, KLRG1 and CD11b suggesting activation state (S1A Fig). Memory NK cells, against haptens and CMV, lack or had decreased CD27 expression [5,22]. Memory like NK expressing CD27 and KLRG1 developed in a bacterial infection model [23]. Memory T cell response is attenuated in the absence of CD27 signaling [24,25]. Thus, we first analyzed response by CD27^+^ NK cells at memory phase (day 37 p.i.) and found increased production of IFN-γ by these cells as compared to naïve CD27^+^ cells (S1B and S1C Fig). The enhanced production of IFN-γ by CD27^+^ NK cells at memory time point prompted us to investigate whether CD27^+^ memory like NK cells are generated after ZIKV infection and whether such cells play a role in antiviral immunity. To establish the role of these cells in antiviral immunity C57BL/6 mice lacking type I IFN receptor (*Ifnar1*), which are highly susceptible to ZIKV, were used [26,27]. The CD27^+^ NK cells purified from naïve C57BL/6 mice and day 37 p.i. (memory phase) were compared for protective efficacy with CD27^-^ NK (memory phase). The transferred cells had no contamination of T lymphocytes. The CD27^+^ NK cells from memory phase significantly reduced viremia (>1log) when transferred to *Ifnar1*^-/-^mice compared to recipients who did not receive any cells (Fig 1A) or recipients of naïve CD27^+^ or CD27^-^ NK cells from memory phase. We next investigated whether superior antiviral ability of memory phase CD27^+^ NK cells was reflected by their higher proliferative capacity. The cell trace violet (CTV) labelled and transferred memory phase CD27^+^ NK cells proliferated and demonstrated up to 4 cell divisions while majority of the CD27^-^ NK cells remained undivided which demonstrates recall potential of memory phase CD27^+^ NK cells (Fig 1B–1E). Moreover, the CD27^+^ NK cells became CD27^-^ (S1D Fig) and gave rise to effector NK cells that degranulated and produced IFN-γ (S1F and S1I Fig). In contrast, non-memory CD27^-^ NK cells did not convert into CD27^+^ NK (S1E Fig) and generated fewer effectors (S1F and S1I Fig).

To investigate whether ZIKV experienced memory phase CD27^+^ NK proliferate in a ZIKV specific manner, memory phase CD27^+^ NK cells were transferred into recipient mice which were challenged either with mock (PBS), ZIKV, flu virus or *E*. *coli*. The CD27^+^ NK cells proliferated in the presence of ZIKV but not with flu virus or *E*. *coli* or in the absence of antigen, indicating the ZIKV specific memory like response (Figs 1F, S1J, S1K and S1L). We also found that memory phase CD27^+^ NK cells incurred lower oxidative damage, demonstrated by decrease in reactive oxygen species (ROS) (Figs 1G, 1H and S2A). The memory phase CD27^+^ NK cells also revealed lesser mitochondrial transmembrane potential dissipation (ΔΨm) (Figs 1I and S2B). On the contrary, CD27^-^ NK cells had higher ROS, high ΔΨm and significantly more cells undergoing apoptotic cell death (S2C Fig). Thus, persistence of ZIKV reactive CD27^+^ NK cells is accompanied by healthy mitochondria and lesser cell damaging ROS. These data together support the notion that CD27^+^ NK cells, probed a month post ZIKV infection, possess memory like features and demonstrate higher antiviral potential than CD27^-^ NK subset.

### NK cells display memory capacity and stem cell-like properties

**Fig 2:**
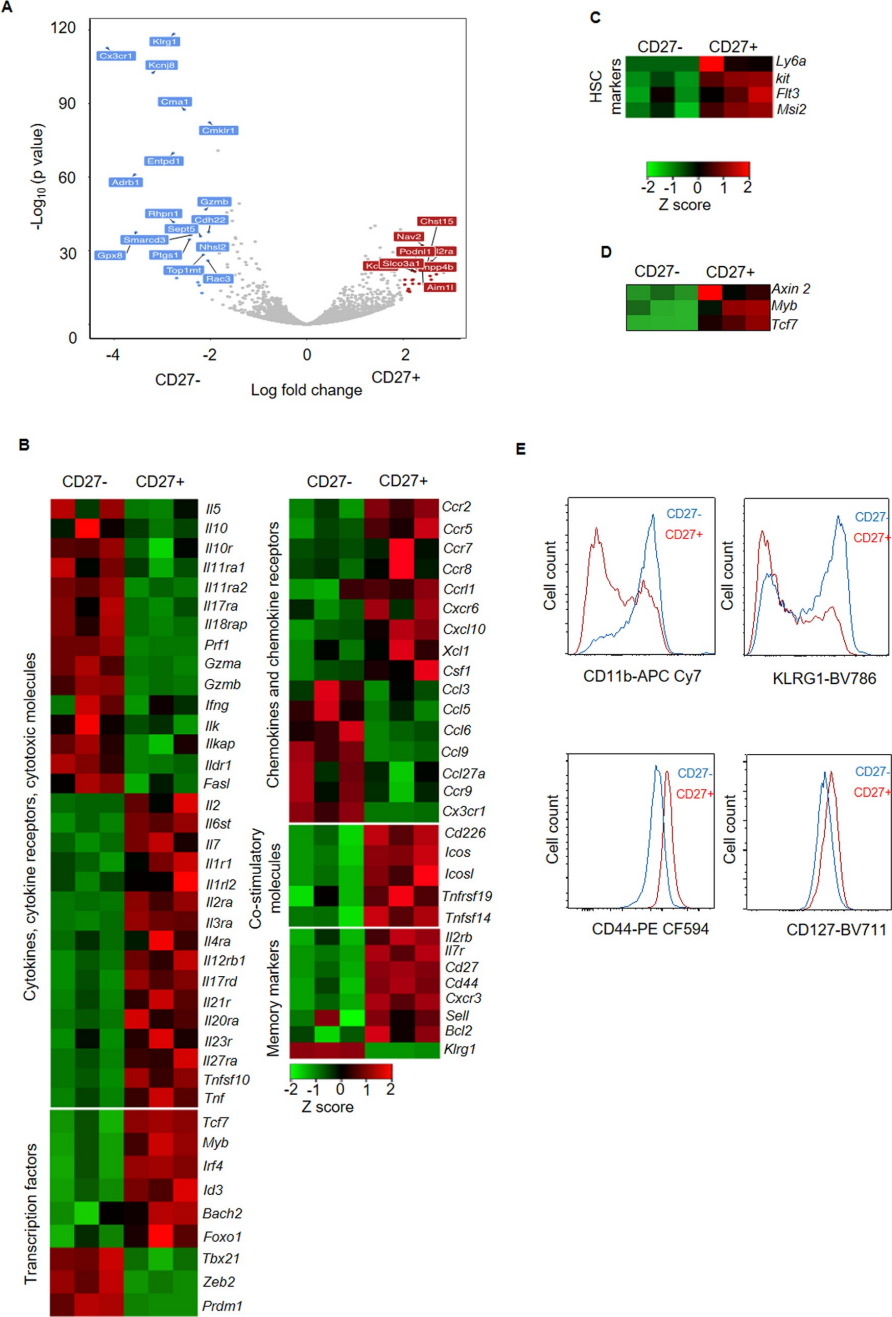
RNA-seq analysis of CD27^+^ memory like NK compared to non-memory CD27^-^ NK cells. (**A**) Volcano plot showing differential genes in CD27^+^ memory like NK compared to non-memory CD27^-^ NK cells a month post ZIKV infection. RNA-seq data are from 3 biological replicates for each group. (**B**) Heat maps of selected molecules such as cytokines, chemokines, co-stimulatory molecules, transcription factors and memory markers in CD27^+^ memory like compared to non-memory CD27^-^ NK cells. (**C**) Heat maps showing selected HSC marker genes in CD27^+^ memory like compared to non-memory CD27^-^ NK cells. Validation of RNA-seq data by assessing selective molecules by qPCR (**D**) and flow cytometry (**E**).

**Fig 3:**
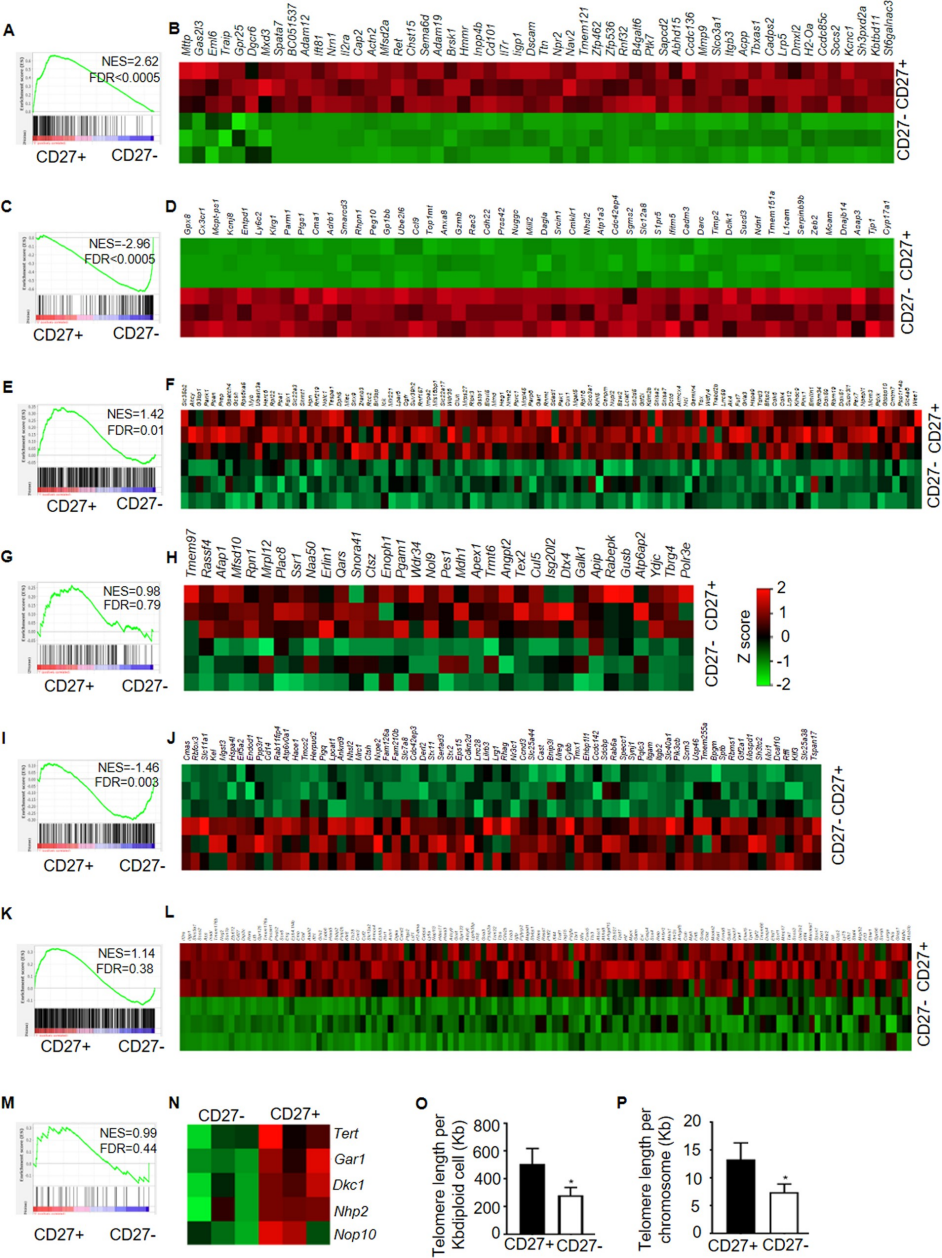
NK cells display memory capacity and stem cell-like properties. RNA-seq (GSE148205) data are from 3 biological replicates for each group. GSEA enrichment plots and leading-edge heat maps for CD8 memory precursor (GSE8678) (**A, B**), CD8 terminal effector (GSE8678) (**C, D**) signatures among memory phase CD27^+^ and CD27^-^ NK gene sets. RNA-seq data are from 3 biological replicates for each group. GSEA enrichment plots and leading edge heat maps for HSC early progenitor (**E, F**), HSC intermediate progenitor (**G, H**), HSC mature cell (**I, J**) and adult tissue-specific stem cell (GSE10423) (**K, L**) signatures among memory phase CD27^+^ and CD27^-^ NK gene sets. (**M**) GSEA enrichment plots comparing telomerase pathway genes in memory phase CD27^+^ compared to CD27^-^ NK. (**N**) Heat map showing relative expression of telomerase complex genes. (**O, P**) Representative telomere length of two biologically independent experiments in memory phase CD27^+^ and CD27^-^ NK cells. Z scores same for all heat maps. Mean ± s.d. two-sided Student’s t-test. *P ≤ 0.05.

To further probe differences in cellular processes, pathways and functionality among CD27^+^ memory like and non-memory CD27^-^ NK cells, we performed RNA sequencing (RNA-seq) analysis. The differences were evident in memory molecules, transcription factors as well as cytokines, chemokines and co-stimulatory molecules (Fig 2A and 2B). The CD27^+^ NK cells showed higher expression of co-stimulatory molecules such as *Cd226* (DNAM-1) (Fig 2B). Gene set enrichment analysis (GSEA) demonstrated memory CD8 T cell genes [28] were over-represented in CD27^+^ NK cells (Fig 3A and 3B) while genetic profile of CD27^-^ NK cells mirrored terminal effectors (Fig 3C and 3D). Furthermore, we noted higher expression of *Foxo1*, *Bach2*, *Id3* and *Irf4*, transcription factors elevated in memory CD8 T cells [28] to be high in CD27^+^ NK cells while expression of *Prdm1* (Blimp1), *Zeb2* and *Tbx21* associated with terminal differentiation and effector function in CD8 T cells [28], remained higher in non-memory CD27^-^ NK cells (Fig 2B). RNA-seq data was validated by assessing expression of selected genes by qPCR and protein expression by flow cytometry (Fig 2D and 2E). Overall, our results indicate parallels among memory CD8 T cells and CD27^+^ memory like NK with higher expression of several transcription factors, previously associated with memory CD8 T cells, in CD27^+^ memory like NK cells.

**Fig 4:**
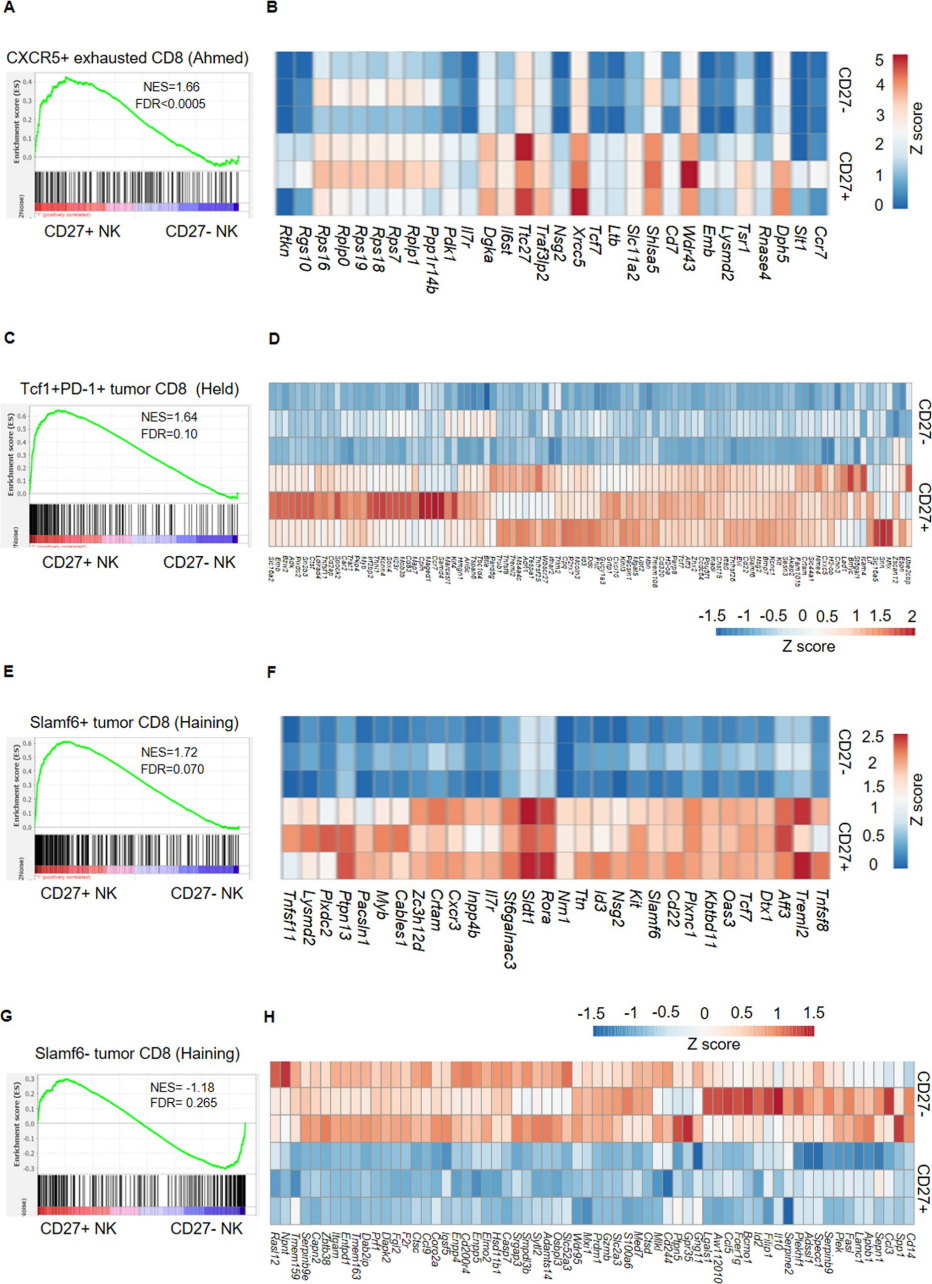
NK_SCM_ cells share gene signature with stem like T cells in chronic viral infection and tumors but differ from exhausted CD8 T cells. GSEA enrichment plots and leading edge heat maps comparing signature of CXCR5^+^ CD8 T cells in chronic LCMV infection (GSE84105) (**A, B**), Tcf1^+^ tumor CD8 T cells (GSE114631) (**C, D**), Slamf6^+^ tumor CD8 T cells (GSE122713) (**E, F**) and Slamf6- tumor CD8 T cells (**G, H**) in CD27^+^ memory like NK cells compared to non-memory CD27^-^ NK cells. RNA-seq data are from 3 biological replicates for each group.

We found that CD27^+^ memory like NK cells had higher expression of *Tcf7* (TCF-1) and *Myb* (cMyb) (Fig 2B) which are important mediators of Wnt/β-catenin signaling essential for maintaining stemness in HSC [29–32] as well as stem like CD8 T cells [9,33]. These observations along with upregulation of HSC marker genes [*Ly6a* (Sca-1), *Kit* (cKit), *Flt3*] and stemness related gene *Msi2* (Fig 2C) led us to investigate whether CD27^+^ memory like NK cells share gene signature with HSC and possess stem cell features. When compared to gene signature of HSC [34], GSEA analysis demonstrated that HSC early (Fig 3E and 3F) and intermediate progenitor genes (Fig 3G and 3H) were enriched in CD27^+^ memory like NK cells. On the other hand, CD27^-^ NK cells mirrored HSC mature cells (Fig 3I and 3J). Moreover, CD27^+^ memory like NK cells also shared genes with adult tissue-specific stem cells [35] (Fig 3K and 3L). The enrichment of telomerase pathway and increased expression of telomerase complex genes was also evident in CD27^+^ memory like NK cells (Fig 3M and 3N) and this was supported by longer telomere lengths observed in CD27^+^ memory like NK cells (Fig 3O and 3P). Furthermore, increased expression of self-renewal genes, pro-longevity genes and repression of anti-longevity genes was also observed in CD27^+^ memory like NK cells (S3A–S3C Fig). These results indicate that CD27^+^ memory like NK cells are enriched for genes expressed in stem cells, have longer telomeres and lower expression of senescence genes. Finally, the transcriptional signature of CD27^+^ memory like NK cells was similar to stem like T cells from chronic infection (Fig 4A and 4B) [11] and tumors (Fig 4C–4F) [14,15] but differed from exhausted CD8 T cells (Fig 4G and 4H). All these results converge to the conclusion that CD27^+^ memory like NK cells have stem cell characteristics.

### Epigenetic features of NK_SCM_

**Fig 5:**
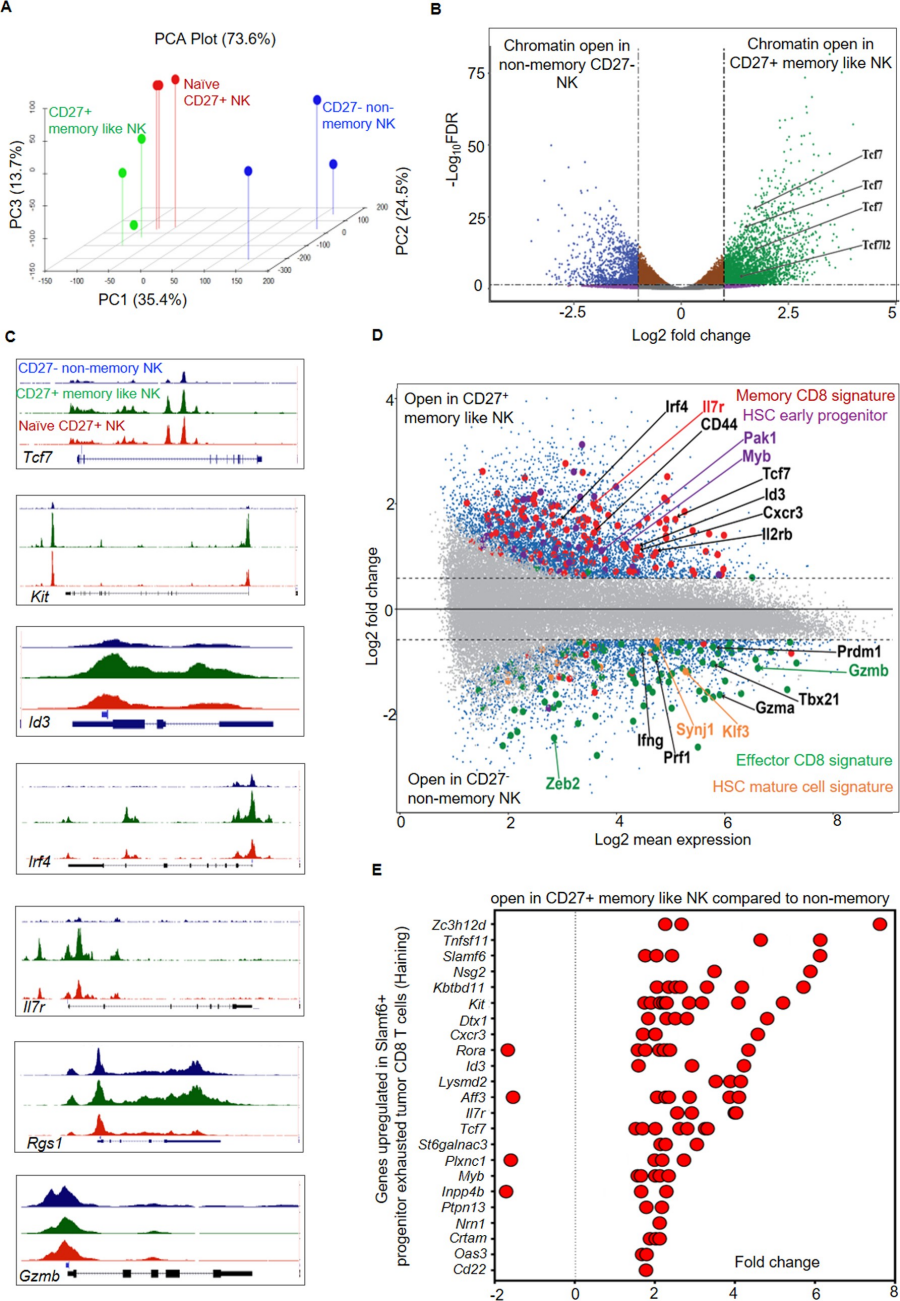
Epigenetic features of NK_SCM_ cells. (**A**) PCA plots for naïve CD27^+^ NK, CD27^+^ memory like and non-memory CD27^-^ NK cells. ATAC-seq data are from 3 biological replicates per experimental group. (**B**) Volcano plot depicting memory like CD27^+^ and non-memory CD27^-^ NK cells showing differently accessible chromatin regions. (**C**) Representative ATAC-seq tracks for the selected loci (*Tcf-7*, *Kit*, *Id3*, *Irf4*, *Il7r*, *Rgs1* and *Gzmb*) in naïve CD27^+^ NK (red), CD27^+^ memory like (green) and non-memory CD27^-^ NK cells (blue). (**D**) MA plot showing differentially accessible (DA) chromatin regions of CD27^+^ memory like and non-memory CD27^-^ NK cells. The dots in blue are significant DA regions. The memory precursor (red) or terminal effector (green) CD8 T cell signature genes and HSC early progenitors (violet) or HSC mature cell (brown) signature genes are shown. DA regions were identified using edgeR. (**E**) Chromatin opening of genes, associated with Slamf6+ progenitor exhausted tumor CD8 T cells, in memory like CD27^+^ NK cells. Fold change values for peaks are plotted in (**E**).

To determine the epigenetic landscape of CD27^+^ memory like NK (d37) and how these cells differ from naïve CD27^+^ NK and non-memory CD27^-^ NK at epigenetic level, we performed genome wide chromatin accessibility analysis using ATAC-seq. The global change in chromatin openness indicates CD27^+^ memory like NK are distinct cells (Figs 5A, 5B and S4A). The major changes in chromatin accessibility were in intronic, intergenic and promoter regions (S4B Fig). We next investigated how CD27^+^ memory like NK cells differed from naïve CD27^+^ NK. We noted 1886 regions differentially accessible in CD27^+^ memory like NK compared to naïve CD27^+^ NK cells (S4A and S5A Figs). Notably, increased accessibility was evident at loci associated with memory such as *Bach2*, *Foxo1*, *Ahr* in CD27^+^ memory like NK cells compared to naïve CD27^+^ NK cells (S5B Fig). The CD27^+^ memory like NK were strikingly different from non-memory CD27^-^ NK (Fig 5A and 5B). ATAC-seq reads revealed differential accessibility at several loci (Fig 5C) with CD27^+^ memory like NK cells showing increased accessibility for memory and stem cell signatures (Fig 5D) on the other hand non-memory CD27^-^ NK cells demonstrated chromatin opening at loci associated with effector T cell and mature HSC signatures (Fig 5D) corroborating our transcriptomics analysis (Figs 2B, 3A–3N and 4A–4F) that CD27^+^ NK cells, probed a month post infection, possess memory and stem cell features. Furthermore, CD27^+^ memory like NK cells revealed increased accessibility at telomerase and pro-longevity genes (S6A Fig) with cells sharing epigenetic changes with tissue-specific adult stem cells [35], HSC markers (S6B and S6C Fig), stem like CD8 T cells from tumors (Figs 5E and S6D) [15] and chronic viral infection (S6E Fig) [11] and TH17 stem cells (S6F Fig) [36]. However, the epigenetic signature differed from exhausted CD8 T cells (S7A Fig). Taken together, we present that CD27^+^ memory like NK cells have a distinct epigenetic profile featuring chromatin openness at loci associated with memory and stemness program.

### TCF/Wnt pathway enrichment in NK_SCM_

**Fig 6:**
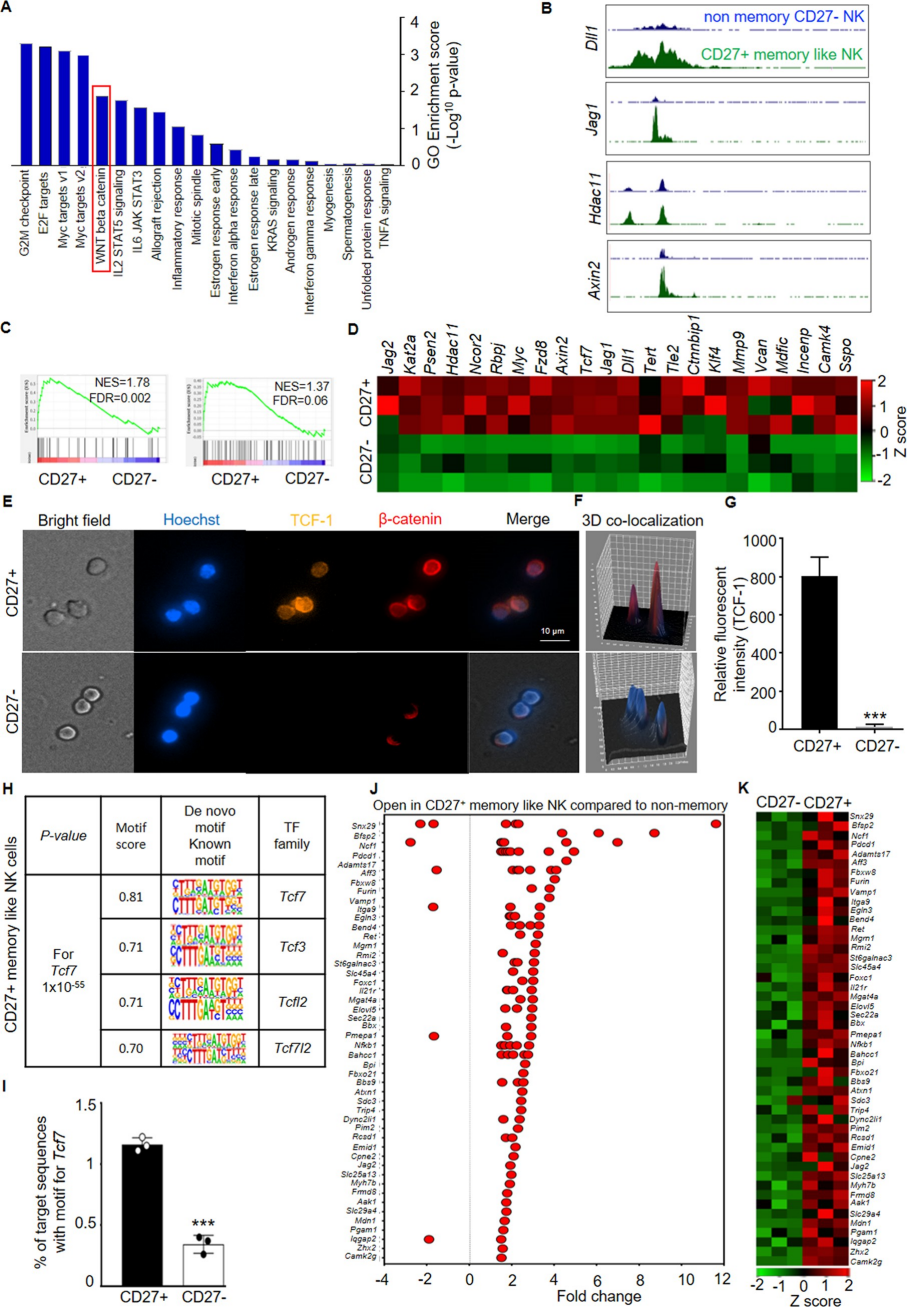
Enrichment of Wnt/β-catenin pathway and active TCF-1 signaling in NK_SCM_ cells. (**A**) Hallmark pathway gene sets analysis in FACS purified CD27^+^ memory like and non-memory CD27^-^ NK cells a month post ZIKV infection. RNA-seq data are from 3 biological replicates for each group. (**B**) ATAC-seq tracks for selected loci of Wnt/β-catenin pathway genes in CD27^+^ memory like (green) and non-memory CD27^-^ NK cells (blue). (**C-D**) GSEA enrichment plots (**C**) and heat maps (**D**) for Wnt/β-catenin pathway (**C left**) and canonical Wnt targets (**C right**) among CD27^+^ memory like and non-memory CD27^-^ NK gene sets. **(E**-**G**) Immunofluorescence images of FACS purified CD27^+^ memory like and non-memory CD27^-^ NK cells, isolated a month post ZIKV infection, and stained for TCF-1 (yellow) and β-catenin (red) show co-localization of both molecules in CD27^+^ cells. Scale bar corresponds to 10 μm. Top-CD27^+^ NK cells, Bottom-CD27^-^ NK cells. Data are representative of 2 independent experiments. (**G**) Relative fluorescent intensity of TCF-1 in CD27^+^ memory like and non-memory CD27^-^ NK. Data are representative of 2 independent experiments (n = 4 per experiment). (**H**) Transcription factor motif enrichment analysis of ATAC-seq from CD27^+^ memory like NK cells. (**I**) % of target sequences with TCF-1 motifs in genomic regions of CD27^+^ memory like and non-memory CD27^-^ NK cells. Chromatin opening by ATAC-seq (**J**) and gene expression by RNA-seq (**K**) of selected TCF-1 target genes in CD27^+^ memory like NK cells. Fold change values for peaks are plotted in (**J**). Mean ± s.d. two-sided Student’s t-test, ***P ≤ 0.001.

To characterize signaling pathways that are unique in CD27^+^ memory like NK, we used hallmark pathway gene sets from mSigDB [37]. The top enriched pathways were associated with active cell cycle (G2M checkpoint, E2F targets, Myc targets) and HSC self-renewal (Wnt/β-catenin pathway) [30–32,38] (Fig 6A). The enrichment of Wnt/β-catenin pathway, transcriptional signature of its targets (Myc, E2F) [38,39] and its role in proliferation of cells [40] suggested a dominant nature of this pathway. GSEA further confirmed enrichment of Wnt signaling genes and canonical Wnt targets in CD27^+^ memory like NK cells (Fig 6C and 6D). This was supported by results from ATAC-seq analysis as open chromatin in Wnt/β-catenin pathway genes were evident in CD27^+^ memory like NK cells (Fig 6B). The CD27^+^ memory like NK cells showed nuclear β-catenin and majority of β-catenin co-localized with TCF-1 by immunofluorescence (Fig 6E and 6F). Moreover, higher protein expression of TCF-1 was also evident in CD27^+^ memory like NK cells (Fig 6G). This was supported by transcription factor motif enrichment analysis which revealed enriched occupancy of factors from the TCF family in CD27^+^ memory like NK cells (Fig 6H). Further, network analysis from these cells revealed that *Tcf7* strongly interacts with several genes associated with Wnt/β-catenin pathway (S6G Fig). We next investigated chromatin accessibility regions of genomes of CD27^+^ memory like and non-memory CD27^-^ NK cells and found increased frequency of *Tcf7* motifs in CD27^+^ memory like NK cells (Fig 6I). Finally, we investigated expression of TCF-1 target genes and found chromatin opening as well as expression of several TCF-1 induced genes in CD27^+^ memory like NK cells (Fig 6J and 6K). Overall, the results indicate that Wnt/β-catenin pathway is enriched and active in CD27^+^ memory like NK cells with these cells also showing enriched occupancy of transcription factors of TCF family.

### TCF-1 is required for memory and stemness program in NK_SCM_

**Fig 7:**
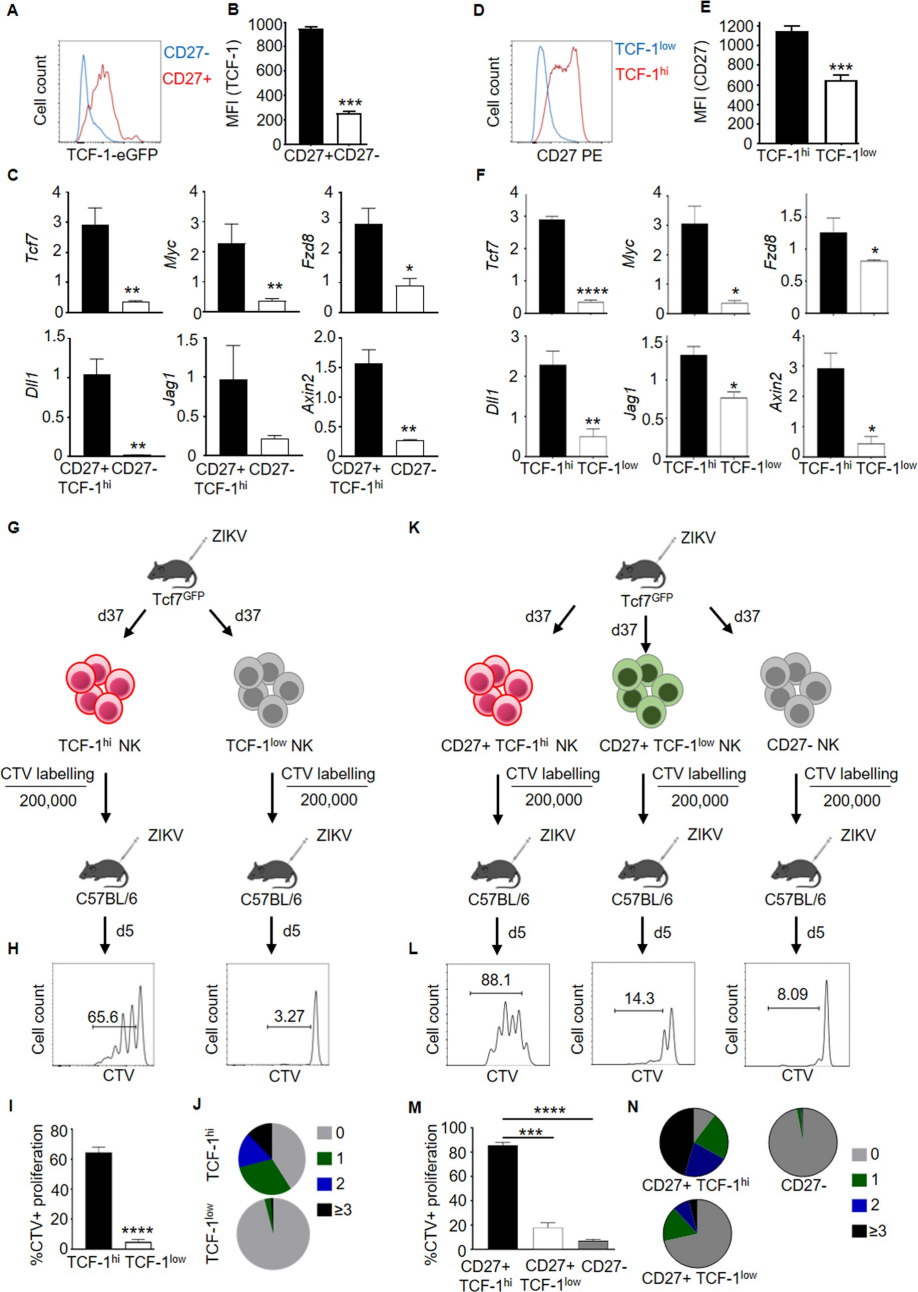
Role for TCF-1 in memory and stemness features in NK_SCM_ cells. (**A, B**) Histogram and MFI depicting TCF-1 expression in CD27^+^ memory like NK and non-memory CD27^-^ NK cells from spleens of *Tcf7*^GFP^ mice a month post ZIKV infection. Data are representative of 2 experiments. (n = 3–4 per experiment). (**C**) Relative expression of Wnt signaling genes in FACS purified CD27^+^ memory like TCF-1^hi^ NK and non-memory CD27^-^ TCF-1^low^ NK cells from spleens of *Tcf7*^GFP^ mice a month post ZIKV infection, using gene specific TaqMan probes. Data are representative of 2 biological replicates (n = 3). (**D, E**) Histogram and MFI depicting expression of CD27 in TCF-1^hi^ and TCF-1^low^ NK cells from spleens of *Tcf7*^GFP^ mice a month post ZIKV infection. (**F**) Relative expression of Wnt signaling genes in FACS purified TCF-1^hi^ and TCF-1^low^ NK cells from spleens of *Tcf7*^GFP^ mice a month post ZIKV infection using gene specific TaqMan probes. Data are representative of 2 biological replicates (n = 3). (**G**) Experimental setup depicting transfer of CTV labelled memory like TCF-1^hi^ NK or TCF-1^low^ NK cells into mice which were challenged with ZIKV and cells were analyzed 5 days later in spleen. (**H-J**) Cellular proliferation of transferred cells is shown. Data are representative of 2 experiments (n = 3–4 per experiment). (**K**) Experimental setup depicting transfer of CTV labelled day 37 p.i. CD27^+^ TCF-1^hi^, CD27^+^ TCF-1^low^ and CD27^-^ NK cells into mice which were challenged with ZIKV and cells were analyzed 5 days later in spleen. (**L-N**) Cellular proliferation of transferred cells is shown. Data are representative of 2 experiments. (n = 3 per experiment). Mean ± s.d. two-sided Student’s t-test, ANOVA. *P ≤ 0.05, **P ≤ 0.01, ***P ≤ 0.001, ****P ≤ 0.0001.

To investigate contribution of TCF-1 in memory and stemness program, *Tcf7*^GFP^ reporter mice were used. We noted that TCF-1 expression was high and contained in majority of CD27^+^ memory like NK cells (Fig 7A and 7B) from *Tcf7*^GFP^ reporter mice while CD27^-^ NK cells were largely negative for TCF-1. To elucidate differences in stemness genes, we purified TCF-1^hi^ CD27^+^ memory like NK and TCF-1^low^ CD27^-^ NK cells from spleen. TCF-1^hi^ CD27^+^ memory like NK cells had significantly higher expression of Wnt signaling genes compared to TCF-1^low^ non-memory CD27^-^ NK cells by qPCR (Fig 7C) suggesting involvement of TCF-1 in stemness. To further demonstrate the role of TCF-1 in driving stemness features in NK cells, memory phase TCF-1^hi^ and TCF-1^low^ NK cells were purified, compared for stemness genes and *in vivo* behavior. The expression of CD27 was high (Fig 7D and 7E) and upregulation of Wnt signaling genes was clearly evident in memory TCF-1^hi^ NK cells (Fig 7F). The memory phase TCF-1^hi^ NK cells proliferated (Fig 7G–7J), converted into TCF-1^low^ cells and gave rise to effectors (S8A–S8E Fig) while TCF-1^low^ NK cells failed to proliferate and convert into TCF-1^hi^ NK cells. Finally, to determine the role of TCF-1 in expansion capacity of NK_SCM_, CD27^+^ TCF-1^hi^, CD27^+^ TCF-1^low^ and non-memory CD27- NK cells were purified from day 37 p.i. *Tcf7*^GFP^ mice, labeled with CTV and were transferred into mice (Fig 7K) which were challenged later with ZIKV. We noted that CD27^+^ TCF-1^hi^ NK_SCM_ robustly proliferated while CD27^+^ TCF-1^low^ NK_SCM_ or CD27^-^ non-memory NK cells failed to significantly proliferate (Fig 7L–7N). Overall, the results suggest cell intrinsic requirement of TCF-1 in memory and stemness features in NK_SCM_ cells.

## Discussion

We provide evidence that NK cells possess memory capacity and stem cell like properties after ZIKV infection. Such NK_SCM_ cells share transcriptional and epigenetic features with stem cells and are similar to exhausted stem like CD8 T cells from chronic infection and tumors. In contrast, NK_SCM_ cells strikingly differ from terminally exhausted CD8 T cells and remain fully functional as shown by their superior antiviral ability compared to naïve or non-memory NK. Epigenetic analysis of NK_SCM_ cells demonstrated enrichment of TCF family transcription factors which likely account for their unusual properties and phenotype.

The NK_SCM_ cells express costimulatory molecule CD27. The CD27 signaling promotes generation of stem cell-like memory T cell subset [25]. It will thus be interesting to determine whether the CD27 signaling cascade is also essential for generating NK_SCM_ and which immune cells provide the stimulatory CD70 ligands. These NK_SCM_ cells also expressed higher levels of another co-stimulatory molecule *Cd226* (DNAM-1) which is of interest since DNAM-1 is required for optimal expansion and differentiation of memory NK against CMV [41]. The functional contribution of DNAM-1 in immune memory exhibited by NK_SCM_ merits further study. Additionally, ZIKV infection upregulates MHC class I molecules on various cell lines which permits evasion of the NK response [42] but NK_SCM_ cells retained antiviral function and protected against ZIKV infection. This could be attributed to accessory help provided by various immune cells and their production of cytokines that act on NK cells as we are further investigating.

Recent studies suggest parallels among memory CD8 T cells and memory NK cells [18,40] a finding supported by our transcriptomics as well as epigenetic data. NK_SCM_ cells exhibited several properties similar to self-renewing HSC’s. First, NK_SCM_ exhibited low ΔΨm indicative of healthy mitochondria and lower amount of oxidative damage as reported for long term persisting stem cells [43]. Second, NK_SCM_ cells have heightened telomerase activity, pro-longevity genes and contain longer telomeres, features commonly observed in stem cells [44]. Such properties likely confer long term persistence of NK_SCM_ cells and multiple rounds of cell division after antigen encounter providing them with durable protective function. It has been reported that the Wnt/β-catenin signaling induces telomerase in stem cells [44]. Our observations of the enrichment of Wnt/β-catenin signaling and increased telomerase expression in NK_SCM_ cells suggest that Wnt signaling might as well induce telomerase expression in NK_SCM_.

We have further demonstrated that the NK_SCM_ cells shared transcriptional as well as epigenetic features with HSC and adult tissue-specific stem cells implicating NK_SCM_ cells contain some common stemness associated pathways. Our data indicates Wnt/β-catenin signaling, associated with HSC self-renewal [30–32,38] as one of the top pathways enriched in NK_SCM_. In line with previous reports [38], our data also revealed nuclear β-catenin as well as its co-localization with TCF-1 further suggesting active Wnt signaling program in NK_SCM_. Many other studies have shown that TCF-1 is required for NK cell development [45,46]. Moreover, our *in vivo* data substantiates the role for TCF-1 in conferring memory and stemness features to NK_SCM_ cells. These data supports the recent report about involvement of Wnt signaling/*Tcf7* in establishment of NK memory in response to HIV induced cytokines [40]. However, the chromatin landscape of NK_SCM_ indicates shared features between NK_SCM_ and stem cells as well as stem like memory T cells implying that the transcription factor TCF-1 confers additional features to NK such as imprinting of stemness program properties. Taken together, we propose that TCF-1 acts as a hub in NK_SCM_ conferring multiple biological functions including memory and stemness program.

Our epigenetic data revealed that, compared to naïve CD27^+^ NK, NK_SCM_ contain open chromatin regions in genes such as *Bach2*, *Foxo1*, *Ahr* which are associated with memory formation [28,47] suggesting acquisition of memory characteristics by NK_SCM_ cells. Our epigenetic results are also in line with reports by others where NK memory against CMV showed differential epigenetic features compared to naïve subsets [18]. A case in point is enrichment of memory and stemness associated open chromatins in NK_SCM_ suggesting possession of both programs. Such memory and stemness features were previously observed in stem like T cells [8,10]. Notably, PD1^+^ CXCR5^+^ CD8 T cells in chronic viral infection [11–13,48,49] and progenitor exhausted CD8 T cells from the tumors [14,15] as well as CD27^+^ Th17 cells from autoimmune disease [36] display stemness. Our data suggest sharedness of transcriptional and epigenetic signature between NK_SCM_ cells and these stem like memory T cells. Several previous publications highlight the importance of TCF-1 in the generation of stem like T cell subset in chronic viral infections [11] as well as in tumors [14,15]. Our data is consistent with these reports.

In conclusion, our results report unique TCF-1^hi^ CD27^+^ NK cells with memory capacity and stem cell features after virus infection. These cells displayed a distinct epigenetic landscape with enrichment of TCF family transcription factors. The transcriptome and epigenome of these cells resembled memory precursor CD8 T cells, HSC, adult tissue-specific stem cells and stem like CD8 T cells from chronic viral infection and tumors. These data support the notion of parallels among memory CD8 T cells and memory like NK with TCF-1 playing a role to maintain both memory and stemness program. The CD27^+^ NK_SCM_ cells can give rise to effectors that produced IFN-γ and these cells had superior ability to control ZIKV compared to non-memory or naïve NK. Thus, seeding of these cells after infection or vaccination can boost antiviral defense. Our results also open up a possibility of existence of NK_SCM_ cells in other infections such as HIV or TB or in the tumor settings. If NK_SCM_ are present in tumors, future experiments should investigate whether those cells respond to PD-1 checkpoint therapy. We speculate beneficial outcome by NK_SCM_ to disrupt tumors. One wonders, however where in the pantheon of antiviral defense mechanisms NK_SCM_ cells feature. We are currently evaluating this issue.

## Materials and methods

### Ethics statement

All animal experiments were performed in accordance with Institutional Animal Care and Use Committee (IACUC) under approved protocols # 635 and 659, and all *in-vitro* experiments and handling of infectious agents were carried out under strict regulation of institutional Infectious Organism Research Review Committee (IORRC) of University of Texas Health Science Center, Tyler (UTHSCT).

### Mice, virus titrations and viral infections

Female 6-8-week-old C57BL/6 mice and breeding pairs for Tcf7^GFP^ and *Ifnar1*^-/-^ mice were purchased from Jackson laboratory. Tcf7^GFP^ and *Ifnar1*^-/-^ mice were bred in house in institutional animal facility. ZIKV strain PRVABC59 (ATCC) was propagated in Vero cell monolayer, titrated and stored in aliquots at -80°C until used.

C57BL/6 and *Tcf7*^GFP^ mice were infected with ZIKV strain PRVABC59 (1×10^7^ PFU, i/p). On day 37 p.i, the mice were sacrificed, and spleen were harvested to assess memory like NK cell responses or to purify NK cell subsets for adoptive transfer. The *Ifnar1*^-/-^ mice were adoptively transferred with various NK populations and infected a day later with 1×10^5^ PFU ZIKV PRVABC59 strain, i/p and viremia measurements were made at day 3 and 5 p.i. C57BL/6 mice were transferred with NK cell subsets and infected intranasally with 10^3^ FFU influenza A virus/Puerto Rico/8/34 (H1N1)(PR8) (Charles River lab)[50] and mice were sacrificed 5 days later.

### Flow cytometry

For surface staining, single cell suspensions from spleen were stained with CD45-BB700 (30-F11+D45, BD Biosciences, #745809), CD3-FITC (145-2C11, BD Biosciences, # 553061), CD3-APC (17A2, BioLegend, #100236), CD3-BV650 (145-C11, BD Biosciences, 564378), NK1.1-FITC (PK136, BD Biosciences, 553164), NK1.1-APC (PK136, BioLegend, 108710), CD27-PE (LG.3A10, BioLegend, #124210), CD-27-PE/Cy7 (LG.3A10, BD Biosciences, #563604), CD44-PE Dazzle (IM7, BioLegend, #103055), CD44-PE-CF594 (IM7, BD Biosciences, #562464), CD44-APC (IM7, BD Biosciences, #559250), CD62L-APC/Cy7 (MEL-14, BD Biosciences, #560514), KLRG1-BV786 (2F1, BD Biosciences, #565477), CD11b-APC/Cy7 (M1/70, BD Bioscience, 557657, Ly49H-BV650 (3D10, BD Biosciences, #744263), CD16/32-APC R700 (2.4G2, BD Biosciences, #565502), DNAM1-Alexa Fluor 647 (10E5, BD Biosciences, #564797), TCF-1 PE (S33-966, BD Biosciences #564217) and NKp46-BV605 (29A1.4, BD Biosciences, # 564069) at 4°C for 30 min, washed with FACS buffer before acquisition and AnnexinV (BD biosciences) in annexin buffer for 10 min.

For intracellular staining, cells were stimulated with PMA and Ionomycin for 4 hrs in the presence of brefeldin (BD Biosciences). After incubation, the cells were surface stained, permeabilized for 30 min at 4°C (BD Cytofix/Cytoperm kit) and stained for intracellular molecules (IFN-γ-BV711, XMG1.2, BD Biosciences, # 564336) for 30 min at 4°C. In CD107a assays, CD107a-APC antibody (1D4B, BioLegend, #121614) was added during stimulation of cells. The cells were acquired in BD LSRFortessa X-20 (BD Biosciences) and data analysis was performed using FlowJo software (TreeStar).

### Cell sorting

Splenocytes were enriched for NK cells using NK cell isolation kit (Miltenyi Biotech) and NK cells were further stained with fluorochrome labelled antibodies (BD Biosciences and BioLegend) and sorted using FACSAria I (BD Biosciences) as indicated in S1J Fig. Purity of the cells obtained in the experiments were >97%.

### Relative quantification of genes by qPCR

RNA was isolated from 50,000 FACS sorted cells using RNeasy Mini Kit (Qiagen) according to manufacturer’s recommendations. The cDNA was synthesized using High-Capacity cDNA Reverse Transcription Kit (Applied Biosystems) and quantification was performed using gene specific TaqMan probes for *Tcf7*, *Myc*, *Fzd8*, *Dll1*, *Jag1*, *Axin2* and *Gapdh*. The relative expression of genes was normalized with *Gapdh*.

### Measurement of viral RNA by qPCR

Serum from infected mice were used for ZIKV RNA isolation as per the manufacturer protocol using Zymo RNA isolation kit (Fisher Scientific). RNA was converted into cDNA and quantification was performed using ZIKV primers and probes by TaqMan method as reported previously [26]. The copy number was determined by standard curve from ZIKV plasmid.

### RNA sequencing and analysis

RNA was isolated from FACS sorted populations (Day 37 CD27^+^ or CD27^-^ NK cells) using NucleoSpin RNA XS columns (Macherey-Nagel, Germany) according to manufacturer’s recommendations. Total RNA was quantified using Qubit fluorometer (Thermo Scientific, USA) and quality was assessed using RNA pico chip (Agilent, USA) on Agilent 2000 Bioanalyzer (Agilent, USA). RNA preparations with RNA integrity number 8 or more were selected for library preparation. The NEBNext Ultra II RNA Library Prep Kit for Illumina (New England Biolabs, Ipswich, MA) was used for generation of RNA libraries according to the manufacturer’s recommendations. Briefly, reverse transcription was performed first to convert RNA to cDNA, followed by end repair, adaptor ligation, and PCR amplification of libraries.

Size estimation of libraries was performed using DNA high sensitivity chip (Agilent, USA) on Agilent 2000 bioanalyzer. The libraries were dual indexed and sequenced on an Illumina HiSeq 4000 NGS System to a depth of 40–50 million single-end 50 bp reads per sample at the Northwestern University NUSeq Core Facility. The reads were trimmed using trim galore and were aligned against mouse reference genome (mm10) using HISAT2. Read counts were estimated using Htseq-counts function and normalization and differential expression analysis were performed using DESeq2.

Publicly available microarray and RNA-seq data were extracted from gene expression omnibus for memory precursor CD8 T cells and terminal effector CD8 T cells (GSE8678), HSC [34], adult tissue-specific stem cells (GSE10423), CXCR5+ CD8 T cells from chronic LCMV infection (GSE84105), Slamf6+ tumor CD8 T cells (GSE122713), tumoral Tcf1+ PD1+ CD8 T cells (GSE114631) and compared with gene expression profiles of different NK cell populations using Gene set enrichment analysis (GSEA). In addition, gene sets hosted at the Molecular Signatures Database for cellular aging and beta-catenin nuclear pathway were also used for GSEA.

### ATAC-seq library preparation

FACS sorted NK cells (Naïve CD27^+^, Day 37 CD27^+^ or Day 37 CD27^-^ NK cells) were counted in TC20 automated cell counter (Bio-Rad, USA) and 50,000 cells were lysed as per standard protocols to isolate intact nuclei and tagmentation reaction was performed using Tagment DNA enzyme I (Illumina, USA) and DNA was purified using Mini elute reaction cleanup kit (Qiagen, Germany). Transposed DNA was amplified using NEBNext High-Fidelity 2X PCR Master Mix (New England Biolabs, USA) and the libraries were purified using Agencourt Ampure XP magnetic beads (Beckman Coulter, USA). Following estimation and quantification, the libraries were sequenced on Illumina Hiseq X to a depth of 50 million paired-end (2x150 bp) reads per sample at Quick Biology Inc, California, USA. The reads were aligned to mouse reference genome (mm10) using Bowtie2 (version 2.1.0) and peak calling was performed using MACS2 in BAMPE mode. Peak differential analysis was performed using edgeR and peaks with fold change greater than 1.5 and FDR < 0.05 were considered as differential peaks.

### Adoptive transfer, labelling with cell trace violet

For adoptive transfer experiments, 2.0×10^5^ NK1.1^+^CD27^+^ or CD27^-^ cells sorted from the spleens of ZIKV infected mice were transferred (i.v.) into naïve or *Ifnar1*^-/-^ mice which were infected a day later with ZIKV. To track proliferation of the cells, sorted CD27^+^ or CD27^-^ NK cells or TCF-1 eGFP^hi^ or TCF-1 eGFP^low^ NK cells or CD27^+^TCF-1 eGFP^hi^ or CD27^+^TCF-1 eGFP^low^ or CD27^-^NK cells were labelled with Cell-trace Violet (C34557, Invitrogen), according to the manufacturer protocol and transferred into mice.

### Immunofluorescence

For immunofluorescence staining, memory like CD27^+^ or non-memory CD27^-^ NK cells were FACS sorted from spleen. The cells were fixed with 4% paraformaldehyde for 15 min at room temperature (RT). Then, cells were washed with PBS containing 0.1% Twin20, pelleted and permeabilized with 1% Twin20 for 30 min at RT. This was followed by washing with PBS containing 0.5% BSA and cells were blocked with PBS containing 0.5% BSA for 1 hr. The cells were pelleted, and 0.5 mg/mL Hoechst dye dissolved in PBS with 0.1% Twin20 was added and cells were incubated for 20 min. Then, cells were washed with PBS containing 0.5% BSA. This was followed by antibody staining step and cells were incubated with TCF-1-PE (4:100) (S33-966, BD Biosciences, #564217) and β-catenin-APC (4:100) (REA480, Miltenyi Biotech, #130124453) antibodies in PBS with 0.5% BSA for 45 min. Then, cells were washed with PBS containing 0.5% BSA, pelleted and subjected to cytospin for 2 minutes and taken on the slides. Finally, the montage media was added, slides were sealed and visualized in Lionheart FX imaging system.

### Mitochondrial potential and ROS measurement

Splenocytes from ZIKV infected mice were used for these ROS and mitochondrial assays. DCFDA staining for ROS was performed as per the manufacturer protocol. Briefly, to measure total ROS, cells were initially surface stained for CD45, CD3, NK1.1 and CD27. Later, the cells were washed and stained for ROS using 1μM of DCFDA (Invitrogen) in PBS and incubated for 5 minutes at 4°C in dark. The cells were immediately acquired in BD LSRFortessa X-20 (BD Biosciences) and data analysis was performed using FlowJo software (TreeStar).

For assessment of mitochondrial membrane potential, the cells were initially surface stained for CD45, CD3, NK1.1 and CD27. Later, the cells were washed and incubated with 50 nM of DiIC1 (Invitrogen) in 1X PBS. The cells were incubated for 15 minutes at 37°C in CO_2_ incubator. The cells were washed twice with 1X PBS and immediately acquired in BD LSRFortessa X-20 (BD Biosciences) and data analysis was performed using FlowJo software (TreeStar).

### Statistical analysis

All experiments were analyzed using GraphPad prism 7 software and R software. The statistical analysis was done using unpaired or paired t test or ANOVA. *P ≤ 0.05, **P ≤ 0.01, ***P ≤ 0.001 and ****P ≤ 0.0001 values were considered significant among groups.

## Supporting information

S1 Fig NK cell activation, IFN-γ production by CD27+ memory like NK cells after Zika virus infection. (A) Expression levels of various molecules on NK cells post ZIKV infection. Data are representative of 2 experiments (n = 3 per experiment). (B, C) IFN-γ production by CD27+ memory like NK compared to non-memory CD27- NK cells. CTV labelled day 37 CD27+ or CD27- NK cells were transferred into mice which were challenged with ZIKV and cells were analyzed 5 days later in spleen. Loss of CD27 (D, E), conversion into effectors (CD44hi NK, IFN-γ+ NK, CD107a+ NK) (F-H) of transferred CD27+ memory like NK or non-memory CD27- NK cells. Data are representative of 2 experiments (n = 3–4 per experiment). (J) Gating strategy to purify NK cell subsets. (K) Gating strategy to analyze CTV+ donor NK in the recipient mice. (L) Cell division of memory phase CD27+ NK transferred into mice which were challenged either with ZIKV, E. coli or no antigen (PBS). Data are representative of 2 experiments (n = 3 per experiment). Mean ± s.d. two-sided Student’s t-test, ANOVA. *P ≤ 0.05, **P ≤ 0.01
(TIF)

S2 Fig CD27+ memory like NK reveal low ROS, lesser loss in mitochondrial membrane potential and cell death. Assessment of ROS (A) by DCFDA staining, mitochondrial membrane potential (B) by DiIC1 assay and cell death (Annexin V+ cells) (C). Data are representative of 3 independent experiments for ROS measurements while representative of two independent experiments for mitochondrial membrane potential and cell death assays (n = 3 per experiment). Mean ± s.d. two-sided Student’s t-test. *P ≤ 0.05, ***P ≤ 0.001.
(TIF)

S3 Fig CD27+ memory like NK cells possess increased expression of self-renewal genes and lower expression of senescence genes. (A) GSEA enrichment plots depicting signature of pro-longevity genes in CD27+ memory like and non-memory CD27- NK cells. (B) Heat map showing expression of aging associated genes among CD27+ memory like and non-memory CD27- NK cells. (C) Heat map showing self-renewal genes among CD27+ memory like and non-memory CD27- NK cells. RNA-seq data are from 3 biological replicates for each group.
(TIF)

S4 Fig Epigenetic landscape of CD27+ memory like NK cells and location of epigenetic changes. (A) Epigenetic landscape of naïve CD27+ NK, CD27+ memory like and non-memory CD27- NK cells. The raw read counts were mean centered and log transformed. Peaks showing no difference between any of the three groups were excluded from the analysis. Selected probes were plotted using a Bioconductor package (ComplexHeatmap Ver 2.2.0). (B) Frequencies of epigenetic change at intronic, intergenic, promoter and exonic regions in CD27+ memory like NK cells. ATAC-seq data are from 3 biological replicates for each group.
(TIF)

S5 Fig CD27+ memory like NK cells differ epigenetically from naïve CD27+ NK cells. (A) Volcano plot depicting differential accessible chromatin regions among naive CD27+ and CD27+ memory like NK cells. (B) Heat maps showing selected open chromatin regions between naive CD27+ and CD27+ memory like NK cells. ATAC-seq data are from 3 biological replicates for each group.
(TIF)

S6 Fig CD27^+^ memory like NK cells reveal chromatin opening for pro-longevity genes and share epigenetic features with stem like T cells. (**A**) Enrichment of chromatin regions of selected pro-longevity genes in CD27^+^ memory like NK cells compared to non-memory CD27^-^ NK cells. Over-representation of chromatin opening for genes associated with adult tissue-specific stem cells (**B**) and heat maps for selected HSC markers (**C**) in CD27^+^ memory like NK cells compared to non-memory CD27^-^ NK cells. Over-representation of chromatin opening for genes associated with Tcf1^+^ PD-1^+^ tumor CD8 T cells (**D**), CXCR5^+^ PD1^+^ CD8 T cells from chronic infection (**E**) and TH17 stem cells from autoimmune disease (**F**) in CD27^+^ memory like NK cells compared to non-memory CD27^-^ NK cells. Fold change values for peaks are plotted in **A**, **B**, **D**, **E** and **F**. ATAC-seq data are from 3 biological replicates for each group. (**G**) Interaction network of TCF-1 with other genes.
(TIF)

S7 Fig CD27+ memory like NK cells differ epigenetically from exhausted CD8 T cells (A) Under-representation of chromatin opening for selected genes associated with nn6- tumor CD8 among CD27+ memory like NK cells and non-memory CD27- NK cells. ATAC-seq data are from 3 biological replicates for each group.
(TIF)

S8 Fig Conversion of memory phase TCF-1hi NK cells into effectors and loss of CD27 and TCF-1 expression. CTV labelled memory like TCF-1hi NK or TCF-1low NK cells were transferred into mice which were challenged with ZIKV and cells were analyzed 5 days later in spleen. (A) Expression of CD44 by transferred donor TCF-1hi and TCF-1low cells. (B) Majority of donor TCF-1hi cells converted into TCF-1low cells. (C) Majority of donor TCF-1low cells remained as TCF-1low cells. (D) Majority of donor TCF-1hi cells converted into CD27 negative cells. (E) Majority of donor TCF-1low cells remained as CD27 negative cells. (Data are representative of 2 experiments. Mean ± s.d. two-sided Student’s t-test. *P ≤ 0.05, **P ≤ 0.01, ***P ≤ 0.001, ****P ≤ 0.0001.
(TIF)
