# Supplementary figures and images for: Memory like NK cells display stem cell like properties after Zika virus infection
Source: PLoS Pathog. 2020 Dec 28;16(12):e1009132. doi: 10.1371/journal.ppat.1009132 (PMC7793296; doi:10.1371/journal.ppat.1009132)

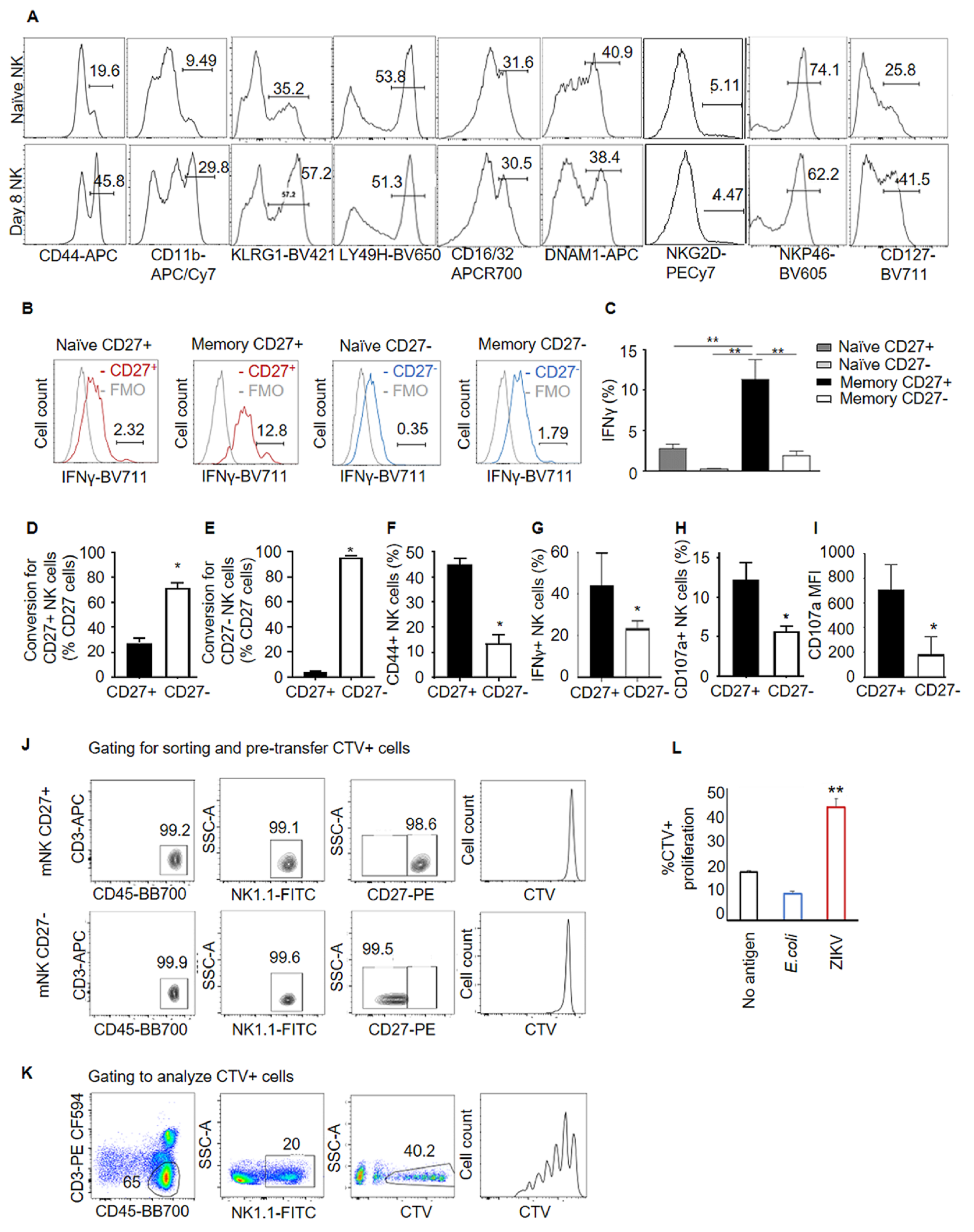

Supplement: S1 Fig — (A) Expression levels of various molecules on NK cells post ZIKV infection. Data are representative of 2 experiments (n = 3 per experiment). (B, C) IFN-γ production by CD27+ memory like NK compared to non-memory CD27- NK cells. CTV labelled day 37 CD27+ or CD27- NK cells were transferred into mice which were challenged with ZIKV and cells were analyzed 5 days later in spleen. Loss of CD27 (D, E), conversion into effectors (CD44hi NK, IFN-γ+ NK, CD107a+ NK) (F-H) of transferred CD27+ memory like NK or non-memory CD27- NK cells. Data are representative of 2 experiments (n = 3–4 per experiment). (J) Gating strategy to purify NK cell subsets. (K) Gating strategy to analyze CTV+ donor NK in the recipient mice. (L) Cell division of memory phase CD27+ NK transferred into mice which were challenged either with ZIKV, E. coli or no antigen (PBS). Data are representative of 2 experiments (n = 3 per experiment). Mean ± s.d. two-sided Student’s t-test, ANOVA. *P ≤ 0.05, **P ≤ 0.01 (TIF) [file ppat.1009132.s001.tif]

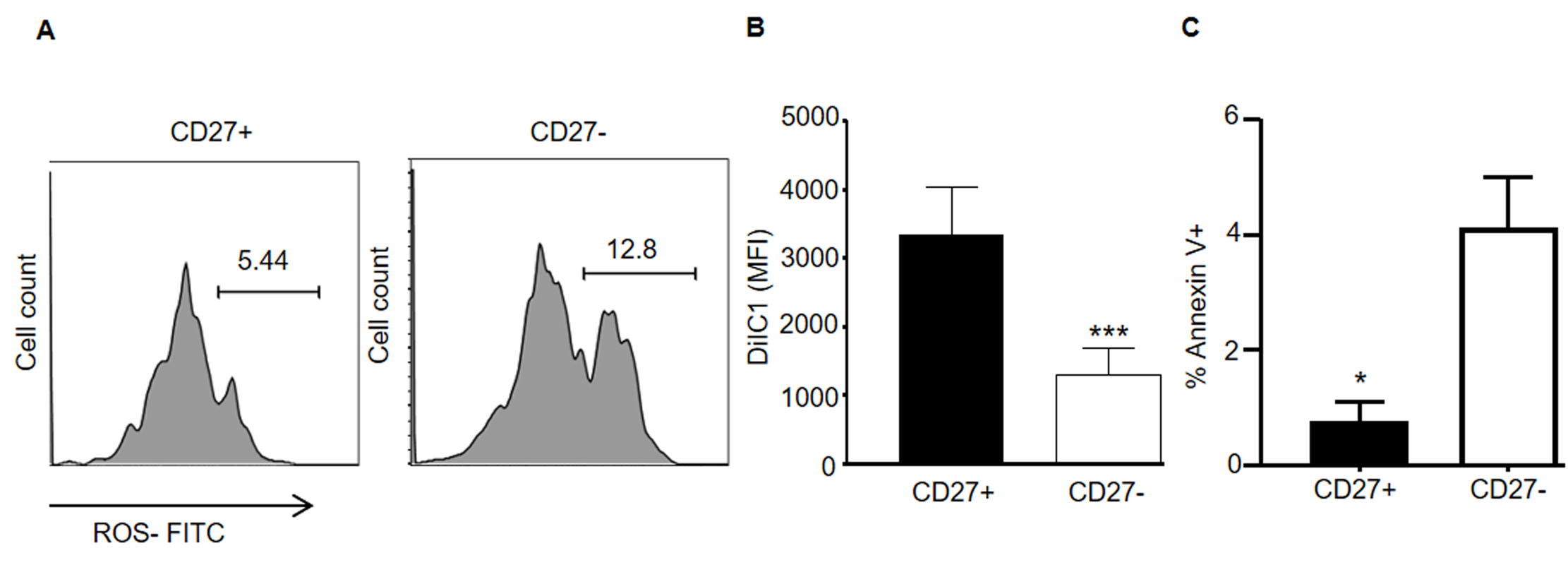

Supplement: S2 Fig — Assessment of ROS (A) by DCFDA staining, mitochondrial membrane potential (B) by DiIC1 assay and cell death (Annexin V+ cells) (C). Data are representative of 3 independent experiments for ROS measurements while representative of two independent experiments for mitochondrial membrane potential and cell death assays (n = 3 per experiment). Mean ± s.d. two-sided Student’s t-test. *P ≤ 0.05, ***P ≤ 0.001. (TIF) [file ppat.1009132.s002.tif]

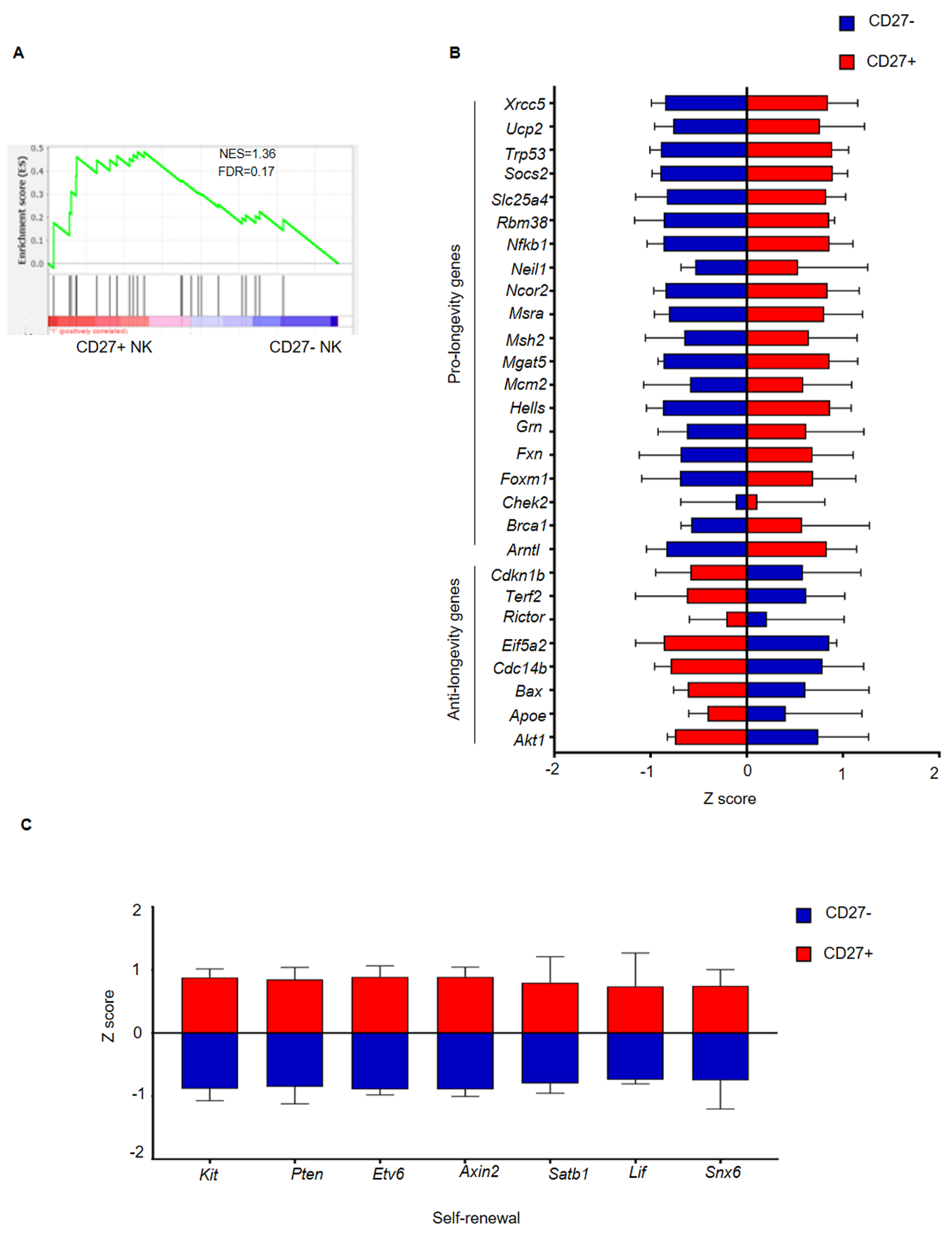

Supplement: S3 Fig — (A) GSEA enrichment plots depicting signature of pro-longevity genes in CD27+ memory like and non-memory CD27- NK cells. (B) Heat map showing expression of aging associated genes among CD27+ memory like and non-memory CD27- NK cells. (C) Heat map showing self-renewal genes among CD27+ memory like and non-memory CD27- NK cells. RNA-seq data are from 3 biological replicates for each group. (TIF) [file ppat.1009132.s003.tif]

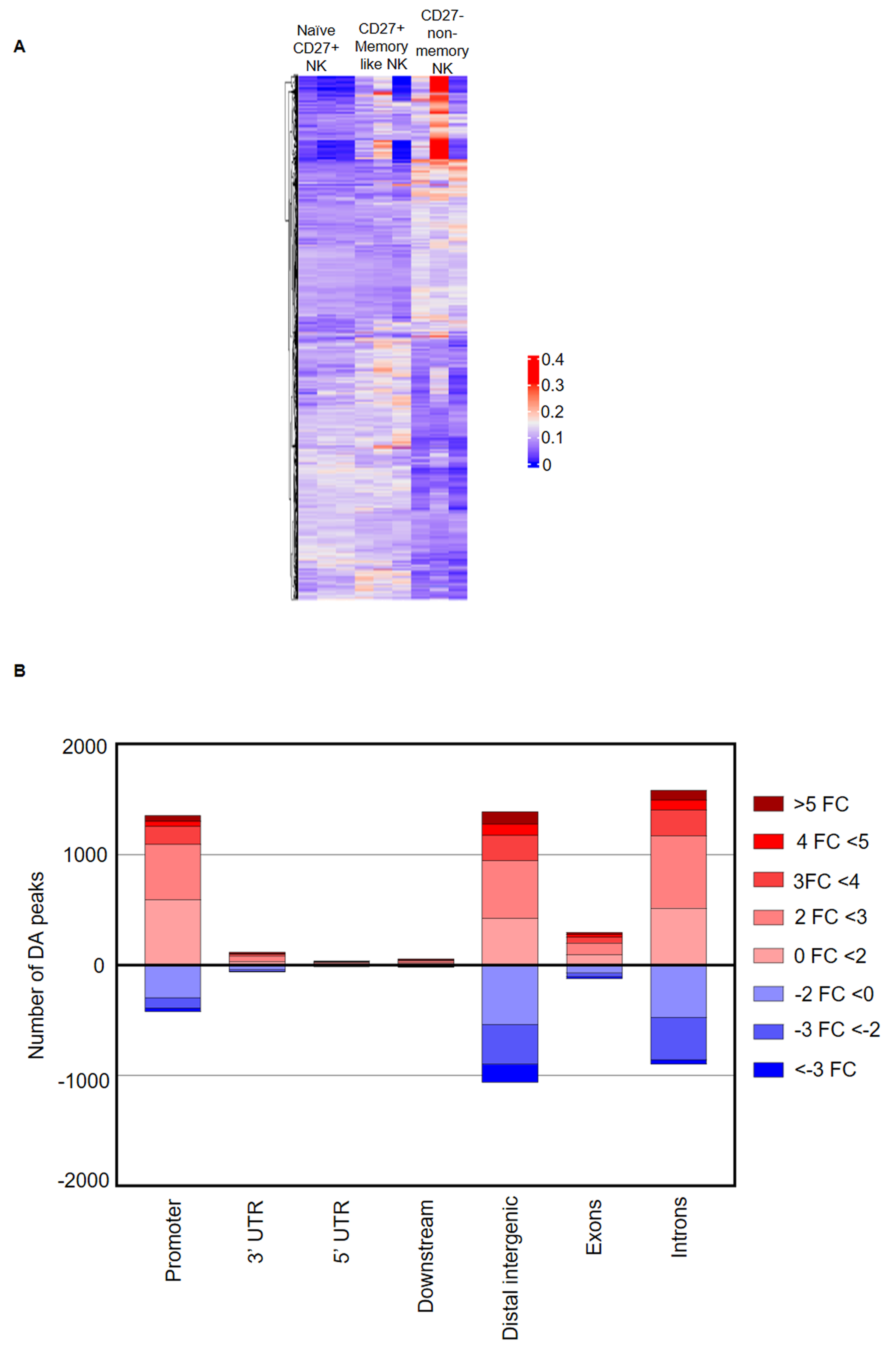

Supplement: S4 Fig — (A) Epigenetic landscape of naïve CD27+ NK, CD27+ memory like and non-memory CD27- NK cells. The raw read counts were mean centered and log transformed. Peaks showing no difference between any of the three groups were excluded from the analysis. Selected probes were plotted using a Bioconductor package (ComplexHeatmap Ver 2.2.0). (B) Frequencies of epigenetic change at intronic, intergenic, promoter and exonic regions in CD27+ memory like NK cells. ATAC-seq data are from 3 biological replicates for each group. (TIF) [file ppat.1009132.s004.tif]

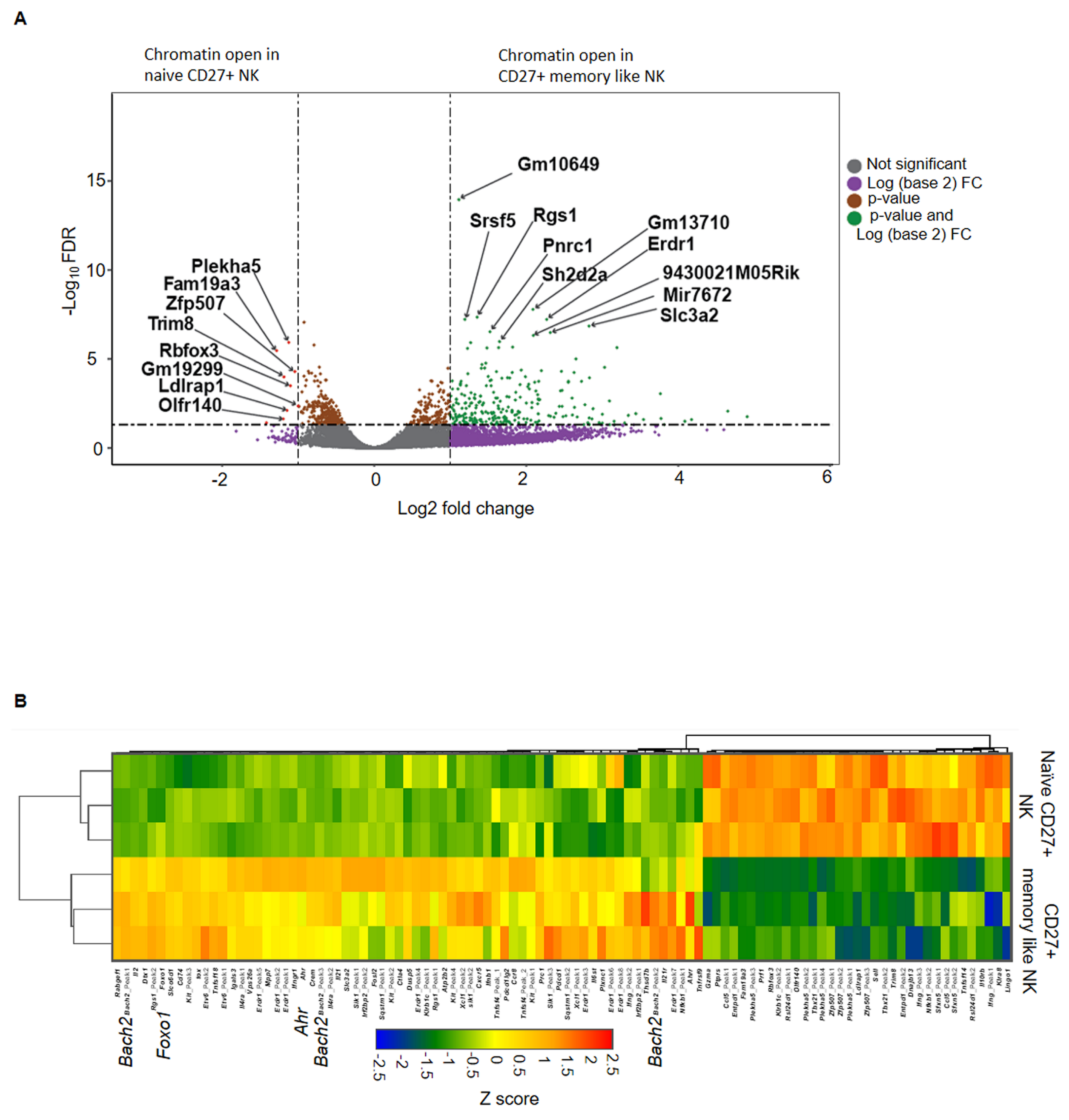

Supplement: S5 Fig — (A) Volcano plot depicting differential accessible chromatin regions among naive CD27+ and CD27+ memory like NK cells. (B) Heat maps showing selected open chromatin regions between naive CD27+ and CD27+ memory like NK cells. ATAC-seq data are from 3 biological replicates for each group. (TIF) [file ppat.1009132.s005.tif]

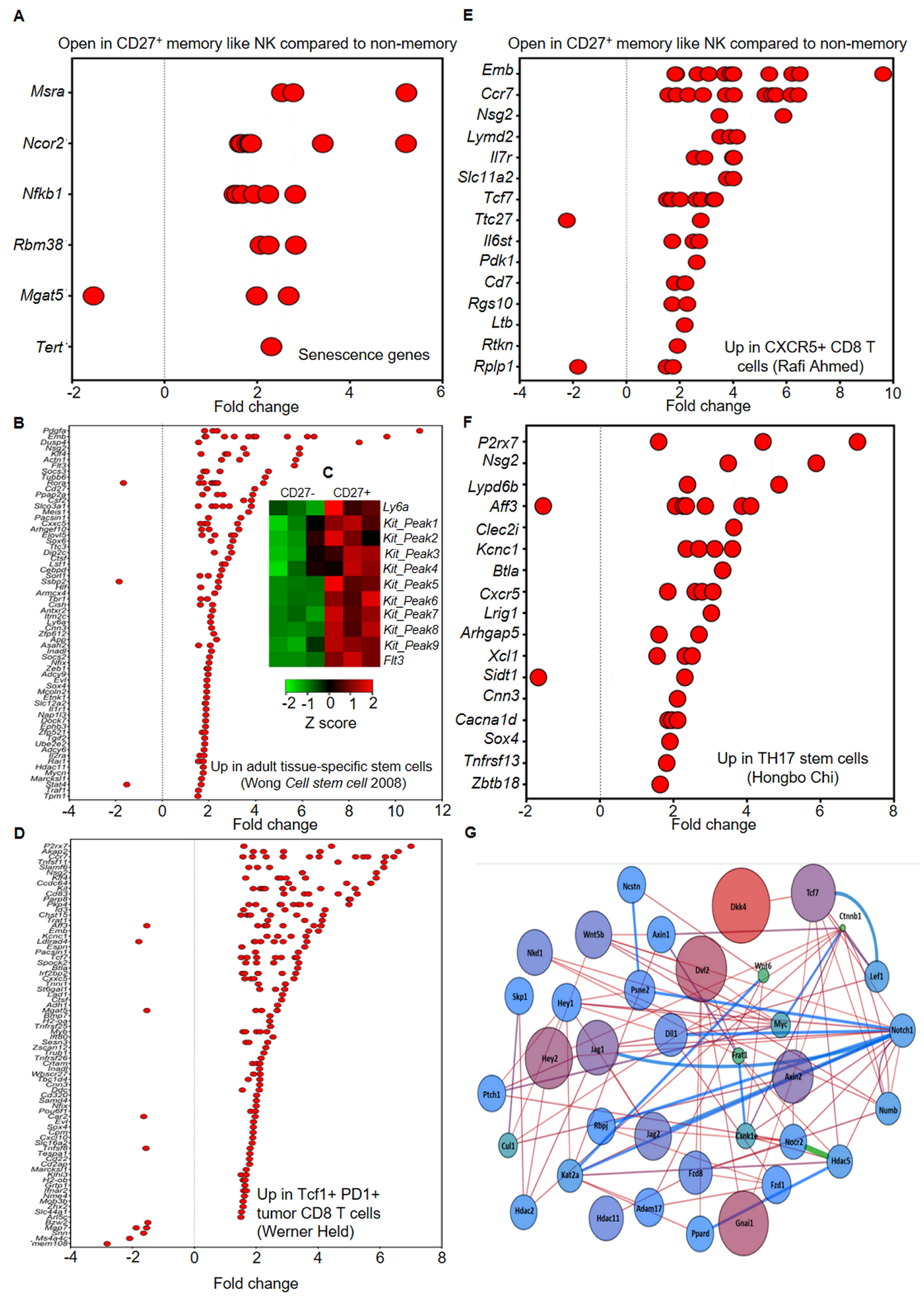

Supplement: S6 Fig — (A) Enrichment of chromatin regions of selected pro-longevity genes in CD27+ memory like NK cells compared to non-memory CD27- NK cells. Over-representation of chromatin opening for genes associated with adult tissue-specific stem cells (B) and heat maps for selected HSC markers (C) in CD27+ memory like NK cells compared to non-memory CD27- NK cells. Over-representation of chromatin opening for genes associated with Tcf1+ PD-1+ tumor CD8 T cells (D), CXCR5+ PD1+ CD8 T cells from chronic infection (E) and TH17 stem cells from autoimmune disease (F) in CD27+ memory like NK cells compared to non-memory CD27- NK cells. Fold change values for peaks are plotted in A, B, D, E and F. ATAC-seq data are from 3 biological replicates for each group. (G) Interaction network of TCF-1 with other genes. (TIF) [file ppat.1009132.s006.tif]

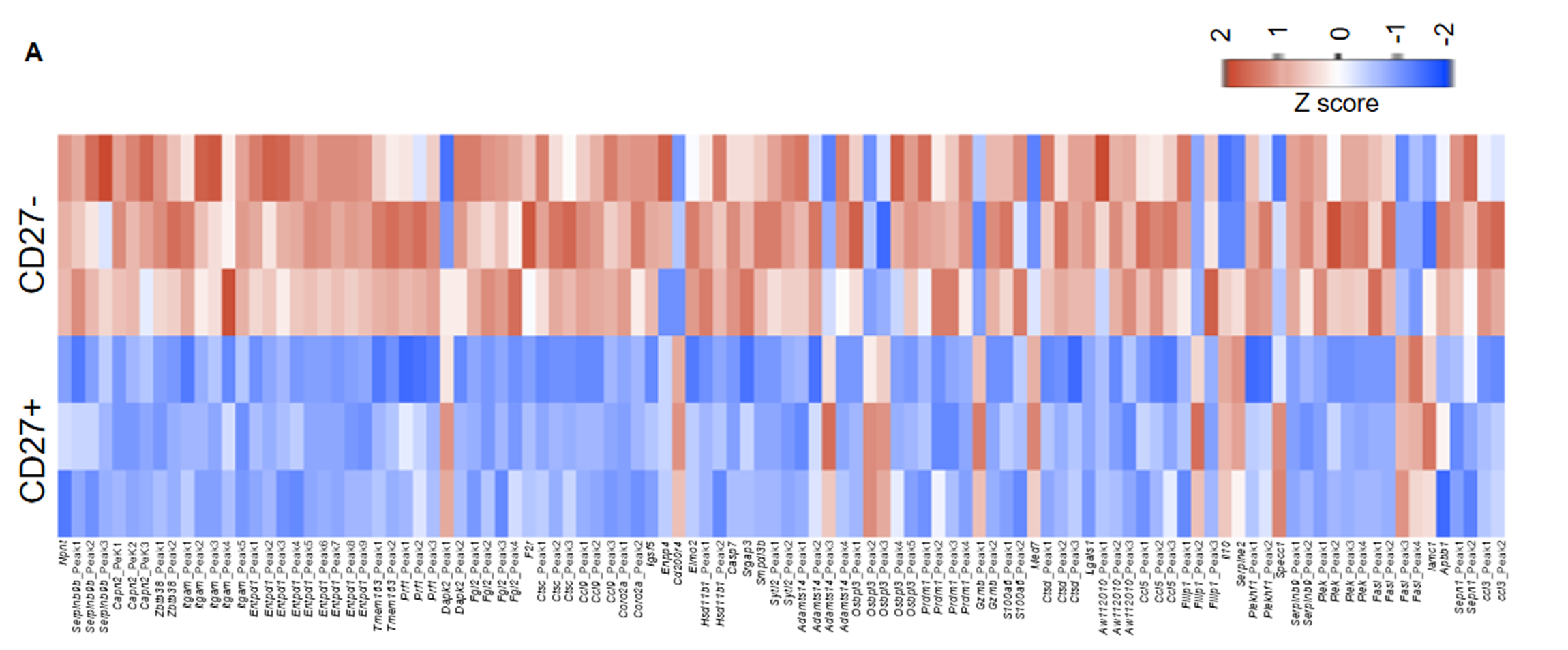

Supplement: S7 Fig — ATAC-seq data are from 3 biological replicates for each group. (TIF) [file ppat.1009132.s007.tif]

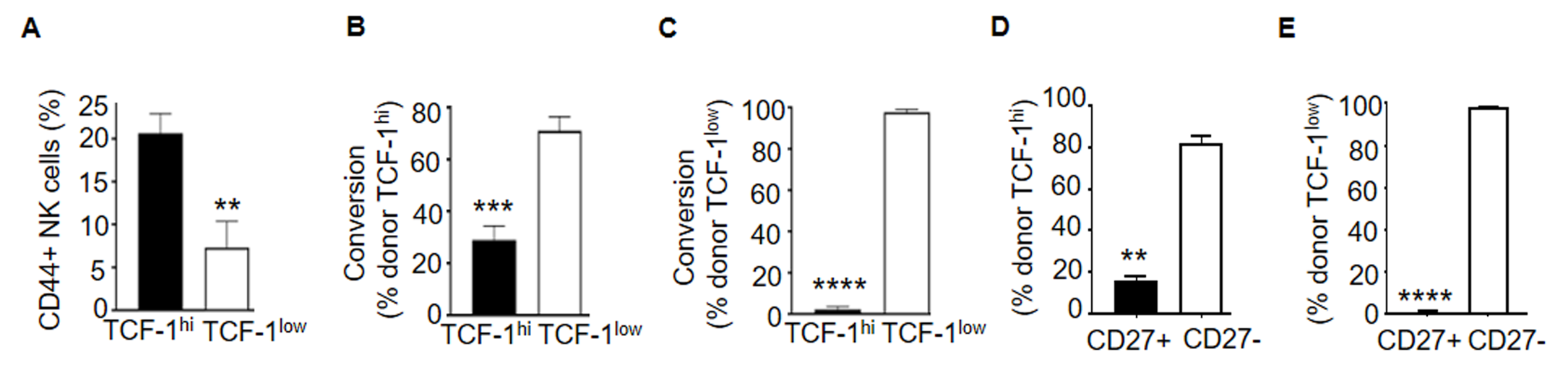

Supplement: S8 Fig — CTV labelled memory like TCF-1hi NK or TCF-1low NK cells were transferred into mice which were challenged with ZIKV and cells were analyzed 5 days later in spleen. (A) Expression of CD44 by transferred donor TCF-1hi and TCF-1low cells. (B) Majority of donor TCF-1hi cells converted into TCF-1low cells. (C) Majority of donor TCF-1low cells remained as TCF-1low cells. (D) Majority of donor TCF-1hi cells converted into CD27 negative cells. (E) Majority of donor TCF-1low cells remained as CD27 negative cells. (Data are representative of 2 experiments. Mean ± s.d. two-sided Student’s t-test. *P ≤ 0.05, **P ≤ 0.01, ***P ≤ 0.001, ****P ≤ 0.0001. (TIF) [file ppat.1009132.s008.tif]
